# Complete magnesiothermic reduction reaction of vertically aligned mesoporous silica channels to form pure silicon nanoparticles

**DOI:** 10.1038/srep09014

**Published:** 2015-03-11

**Authors:** Kyoung Hwan Kim, Dong Jin Lee, Kyeong Min Cho, Seon Joon Kim, Jung-Ki Park, Hee-Tae Jung

**Affiliations:** 1 Department of Chemical and Biomolecular Engineering (BK21+Program), Korea Advance Institute of Science and Technology (KAIST), Daejeon 305-701 (Korea)

## Abstract

Owing to its simplicity and low temperature conditions, magnesiothermic reduction of silica is one of the most powerful methods for producing silicon nanostructures. However, incomplete reduction takes place in this process leaving unconverted silica under the silicon layer. This phenomenon limits the use of this method for the rational design of silicon structures. In this effort, a technique that enables complete magnesiothermic reduction of silica to form silicon has been developed. The procedure involves magnesium promoted reduction of vertically oriented mesoporous silica channels on reduced graphene oxides (rGO) sheets. The mesopores play a significant role in effectively enabling magnesium gas to interact with silica through a large number of reaction sites. Utilizing this approach, highly uniform, *ca.* 10 nm sized silicon nanoparticles are generated without contamination by unreacted silica. The new method for complete magnesiothermic reduction of mesoporous silica approach provides a foundation for the rational design of silicon structures.

Above its melting temperature (T_m_ *ca.* 650°C), magnesium promotes the transformation of micro-scaled solid silica bulk structures into nanostructured silicon (equation 1)^1^. This method contrasts to the conventional carbothermal reduction of silica to form silicon^2^, which operates at temperatures above 2000°C. The silicon produced by using magnesiothermic reduction at 650°C possesses the same 3D structure of the original diatom because the melting temperature of silicon is *ca.* 1414°C. As a result, various, reaction template dependent shapes of silicon nanostructures can be prepared by employing this approach^1^,^3^. Silicon produced by magnesiothermic reduction has been used in a number of applications such as gas sensors^1^, optical devices^4^, optoelectronic devices^5^ and Li-ion batteries^6^,^7^,^8^,^9^.
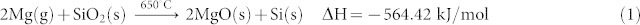


However, generation of silicon via magnesiothermic reduction has a critical limitation caused by unavoidable incomplete reduction that results in the formation of unreacted silica or magnesium silicide. The cause of this phenomenon is that gaseous magnesium reacts with silica on the surface and, consequently, the formed silicon product prevents access of magnesium to silica in the interior. In addition, the presence of unreacted silica causes a mismatch of the stoichiometric ratio of magnesium and silica, which results in an undesired side reaction that produces magnesium silicide (Mg_2_Si, equation 2). Because the presence of unreacted silica and magnesium silicide seriously deteriorates the purity of silicon nanostructures, a higher degree of control over the magnesiothermic reduction reaction is required when high quality silicon nanostructures are desired for practical applications^10^ Although it is known that purity of the silica can be improved by controlling the magnesium to silica ratio^11^ and using temperature ramping^3^, these techniques are still insufficient to fabricate high quality silicon nanostructures. Because of this problem, magnesiothermic reduction is often followed by an additional etching process using hydrofluoric acid (HF). However, while producing more pure form of the material the etching step also leads to serious deterioration and changes in the structure and morphology of the target silicon nanostructures^12^.
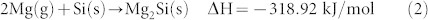


In the study described below, we developed a new approach for the complete conversion of silica which employs magnesiothermic reduction using vertically oriented mesoporous silica channels present in two dimensional materials such as reduced graphene oxide (rGO) sheets. In this system, gaseous magnesium is able to gain access to the silica within the thin films in the channels. By utilizing this approach, we were able to produce *ca.* 10 nm scale silicon nanoparticles that do not contain unreacted silica and undesired magnesium silicide. The superiority of the new technique was demonstrated by its employment to fabricate silicon electrodes in a lithium-ion battery that have good cycling stability.

## Results

**Figure 1:**
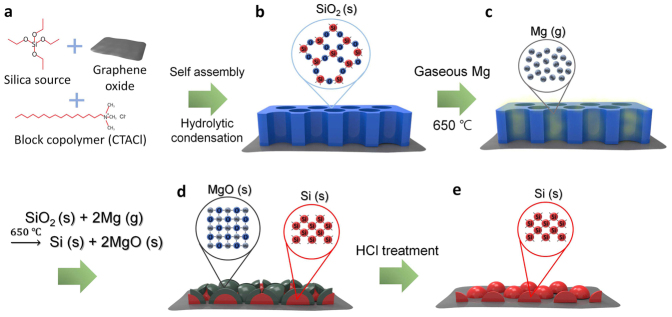
Schematic diagram of the fabrication process. (a) Mixing of silica source, block copolymer and graphene oxides (b) Synthesis of vertically oriented mesoporous silica channels on two-dimensional substrates. (c) Infiltration of gaseous magnesium into mesoporous silica. (d) Formation of magnesium oxide and silicon nanoparticles. (e) The formation of completely reduced silicon nanoparticles after removal of magnesium oxide by treatment with hydrochloric.

The new, mesoporous silica channel based strategy for magnesiothermic reduction of silica to produce silicon is illustrated in Figure 1. In this process, vertically aligned mesoporous silica channels are generated on a two dimensional GO substrate. While GO was used in this study, depending on target applications different types of substrates can be employed for this purpose. The mesoporous silica layer was then formed by simply mixing a solution of the GO substrate with a solution of cetyltrimethylammonium chloride (CTACl) in 1 M NaOH, followed by addition of tetraethyl orthosilicate (TEOS) (Figure 1a)^13^. By using this approach to control the pH precisely at 11.7, the mesoporous silica structure are produced via soft-templating of the block copolymer CTACl followed by hydrolytic condensation with TEOS (Figure 1b). The mesoporous silica formed in this manner was blended with the magnesium granules, placed within an alumina crucible, and heated in a tube furnace at 650°C under an atmosphere of argon (500 sccm) and hydrogen (50 sccm). In this process, magnesium infiltrates into the pores and covers the surface of the mesoporous silica template (Figure 1b), where it promotes the magnesiothermic reaction (equation 1) to produce silicon and magnesium oxide (Figure 1c). Loss of oxygen from silica enables the silicon atoms to arrange into a crystalline phase^1^. Finally, magnesium oxide generated in the reduction reaction is removed by using 1 M hydrochloric acid (Figure 1d). Vacuum filtration then leads to isolation of a dark brown silicon nanoparticle powder that does not contain any unreacted silica (Figure 1e).

**Figure 2:**
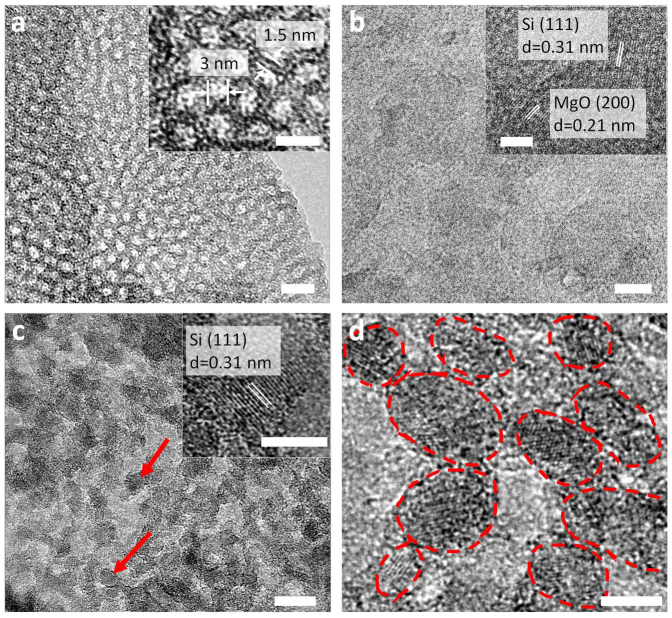
Conversion of vertically aligned mesoporous silica channels to silicon nanoparticles. (a) TEM image of mesoporous silica; scale bar 10 nm. (inset: high magnification image indicating the sizes of pores and walls; scale bar 5 nm); (b) TEM image of the mesoporous silica after magnesiothermic reduction; scale bar 10 nm. (inset: high magnification image indicating the (111) lattice of silicon and the (200) lattice of magnesium oxide (MgO); scale bar 5 nm) (c) TEM image of pure silicon nanoparticles; scale bar, 10 nm. (inset: high magnification image of the (111) lattice spacing of crystalline silicon of pure silicon nanoparticles; scale bar 5 nm.) The red arrow indicates the location of a silicon nanoparticle; scale bar 10 nm. (d) High magnification TEM image of pure silicon nanoparticles showing *ca.* 10 nm size; scale bar 5 nm.

Representative transmission electron (TEM) images of the mesoporous silica on a GO substrate prior to magnesiothermic reduction are displayed in Figures 2a and 2b and those of the resulting silicon nanoparticles are shown in Figures 2c and 2d. In these images, bright and dark areas correspond to the GO substrate and thin walls of the silica channels, respectively. As can be seen, the cylindrical mesoporous silica channels are vertically aligned with respect to the GO substrate (Figure 2a and see Supplementary Fig. S1 online). A magnified image given in the inset of Figure 2a shows the presence of hexagonally ordered silica mosopores with an average pore size of *ca.* 3 nm (standard deviation of pore size, σ = 0.32) and a wall thickness of *ca.* 1.5 nm, determined by averaging the results from analysis of approximately 50 silica pores taken from the TEM images. This finding is consistent with the d_100_ spacing obtained by small angle X-ray scattering (SAXS) analysis (see Supplementary Fig. S2 online). The presence of a strong XRD peak at 2θ = 1.98° indicates a d_100_ spacing of 4.5 nm for the center to center distance of hexagonally ordered silica pores including the pore diameter and wall thickness. Upon magnesiothermic reduction at 650°C, the mesoporous silica transforms into silicon nanoparticles covered with magnesium oxide. Inspection of a high magnification image (see Supplementary inset of Fig. 2b online) shows the presence of lattice images for silicon and magnesium oxide. The lattice fringe with 0.31 nm spacing corresponds to the (111) plane of silicon, while that with 0.21 nm spacing is associated with the (200) plane of magnesium oxide. After removal of magnesium oxide by using hydrochloric acid, only the silicon nanoparticles remain well-distributed and without considerable aggregation on the rGO substrate (Figure 2c). The nanoparticles have mostly a spherical shape and a mean diameter of *ca.* 10 nm (standard deviation, σ = 2.4), as determined by averaging measurements of approximately 200 silicon nanoparticles taken from different TEM images. The TEM images show that only silicon nanoparticles exist in the entire specimen (inset of Figure 2c), suggesting that complete reduction has taken place and that undesired products such as magnesium oxide or magnesium silicide are not present. It is important to note that the silicon nanoparticles prepared by using the new approach are both very small and uniform, features that contrasts with those prepared by utilizing previous magnesiothermic reduction reactions that have a *ca.* 30 nm size^12^. This result is attributed to the existence of vertically oriented mesoporous silica channels, which increase the accessibility of gaseous magnesium to silica during the reduction process. Also, the thin walls of the silica channels (*ca.* 1.5 nm) prevent severe aggregation of silicon atoms.

**Figure 3:**
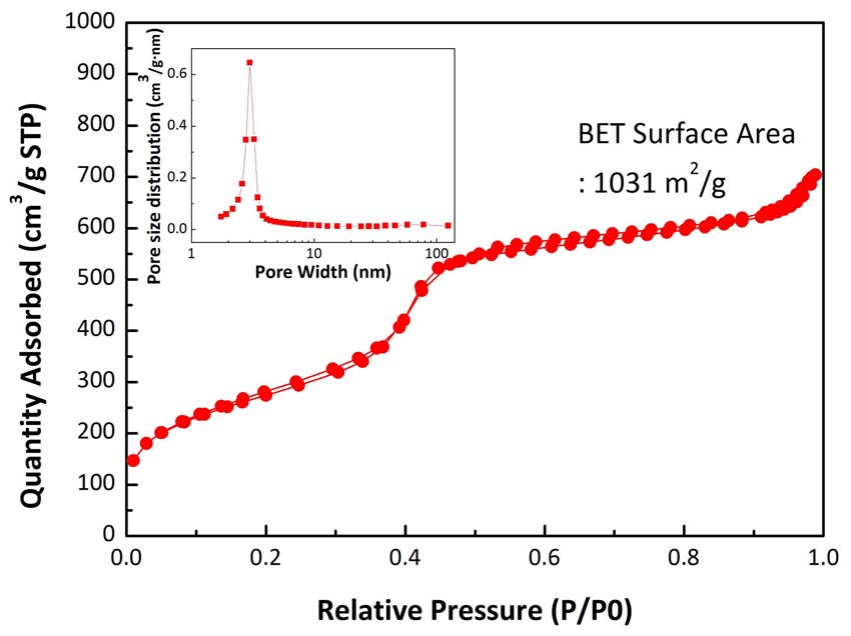
N_2_-sorption analysis of vertically aligned mesoporous silica channels. N_2_-adsorption-desorption isotherms of vertically aligned mesoporous silica channels (inset: pore size distributions).

The specific surface area and pore size of the mesoporous silica were determined by employing nitrogen-sorption analysis at 77 K (Figure 3). The adsorption-desorption isotherm exhibits a typical type IV isotherm for the N_2_-sorption branch with an pronounced adsorption at *ca.* 0.4 P/P_0_, indicating that uniform cylindrical mesopores are present. The pore size distribution is calculated using the adsorption branch of the capillary condensation region utilizing the Barrett-Joyner-Halenda (BJH) algorithm^14^. The results show that a narrow distribution of mesopore diameters centered at 3 nm are present (inset of Figure 3), which is consistent with the pore size measured from the TEM image (Figure 2a). Also, the adsorption and desorption branches display very small hysteresis, demonstrating that the mesopores are uniform and open without any pore-blocking effects occurring during desorption^15^. Consequently, the specific surface area of the vertically aligned mesoporous silica, calculated by using the Brunauer-Emmett-Teller method^16^ is 1030 m^2^/g, is considerably higher than that of previously reported mesoporous silica^13^,^17^,^18^. The adsorption-desorption isotherm of the silicon nanoparticles resulting from the magnesiothermic reduction reaction displays small hysteresis of type H3 at 0.4-0.8 P/P_0_ (see Supplementary Fig. S3 online), indicating plate-like particles that do not display pore-blocking effects during desorption^15^. The specific surface area of the completely reduced silicon nanoparticles is 363 m^2^/g, a value that is large as compared to that obtained in previously generated silicon nano-structures produced by using magnesiothermic reduction^19^,^20^,^21^. This outcome is the consequence of the fact that the starting silicon particles with *ca.* 10 nm size are well distributed over the rGO sheets without significant agglomeration or a restacking of rGO sheets.

**Figure 4:**
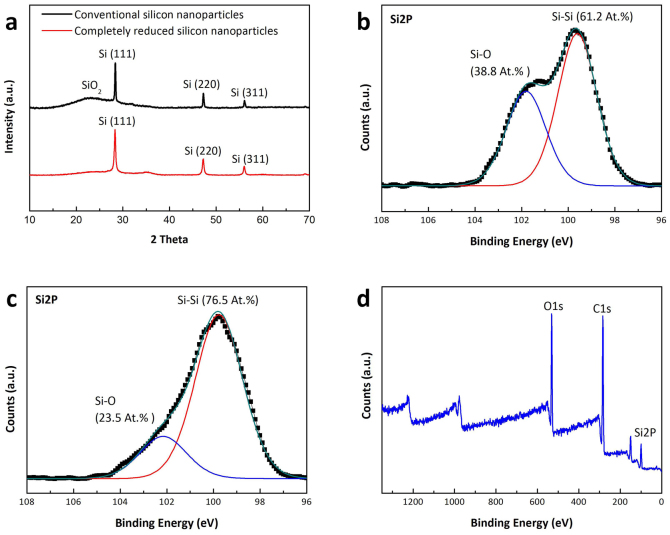
Characterization of completely reduced silicon nanoparticles. (a) Wide-angle X-ray scattering (WAXS) patterns of completely reduced silicon nanoparticles (red line), and conventional silicon nanoparticles (black line). X-ray photoelectron spectroscopy (XPS) spectrum of Si2p of (b) conventional silicon nanoparticle, (c) completely reduced silicon nanoparticles (d) the survey spectrum of completely reduced silicon nanoparticles.

To verify that complete conversion of silica to silicon takes place in the magnesiothermic reduction carried out by using the new approach with vertically aligned mesoporous silica channels, analyses of wide angle X-ray diffraction (XRD) pattern and X-ray photoelectron spectroscopy (XPS) spectra were performed. For this purpose, XRD of silicon nanostructures were prepared by magnesiothermic reduction reactions of two different silica templates. First, silicon nanoparticles were generated by employing a conventional silica-GO composite as a control (black line). In this case, XRD analysis (Figure 4a) shows that the silica layer not having a porous structure is formed on the graphene oxide sheets by simply mixing of TEOS and GO^12^. Second, silicon nanoparticles were prepared by using the mesoporous silica structure (red line). Analysis of the XRD patterns of the silicon particles produced by reduction of the conventional silica composite show three prominent diffraction peaks for silicon at 2θ = 28.4°, 47.3° and 56.1° (JCPDS #.65-1060), along with a broad peak around 2θ *ca.* 23° corresponding to residual silica. In contrast, the XRD pattern for silicon nanoparticle derived from the mesoporous silica only contains diffraction peaks for silicon only.

In order to further demonstrate that complete magnesiothermic reduction takes place when vertically aligned mesoporous silica samples are employed, the chemical bonding status of silicon was elucidated by using X-ray photoelectron spectroscopy (XPS). The Si2p spectrum of the silicon particles arising from silica composite produced using the conventional method (Figure 4b) contains two peaks at 99.5 eV and ~102.2 eV, corresponding to Si-Si (elemental silicon) and Si-O (silicon oxide) bonds, respectively^22^,^23^,^24^. The existence of a strong peak for Si-O bond (38.8%) in this spectrum indicates the presence of silica residues. In contrast, the Si2p spectrum of completely reduced silicon nanoparticle formed by using the new strategy (Figure 4c) contains mainly a peak for Si-Si bonds at 99.5 eV. However, deconvolution of the Si2p XPS peak of the spectrum of completely reduced silicon nanoparticles shows that a Si-O peak (23.5%) exists, although it has a much reduced intensity as compared to silicon nanoparticles prepared by using a conventional silica composite. To show that the relatively weak Si-O peak does not arise from residual silica in the reduction reaction but rather from Si-O naturally formed on the surface of silicon nanoparticles after reduction^25^, we examined Si-O peak of the silicon nanoparticles prepared by using a conventional silica composite after removing the residual silica by HF treatment of silicon particles. The shape and ratio of the Si-O peak for the sample after HF treatment are almost identical to those of the silicon nanoparticles prepared by using mesoporous silica structures and not treated with HF (see Supplementary Fig. S4 online). This observation indicates that Si-O in the XPS of the mesoporous structure originates from naturally formed silica layer, not from residual silica. In fact, it has been demonstrated previously that a thin layer of silica is naturally formed on silicon surface in the presence of air and humidity at room temperature^25^,^26^. Finally, inspection of the XPS spectrum of completely reduced silicon nanoparticles on rGO (Figure 4d) shows that no impurities such as Mg_2_Si or Mg_2_SiO_4_ are present and that only carbon (C1s), silicon (Si2p) and oxide (O1s) atoms exist, which arises from rGO and silicon nanoparticles, respectively.

**Figure 5:**
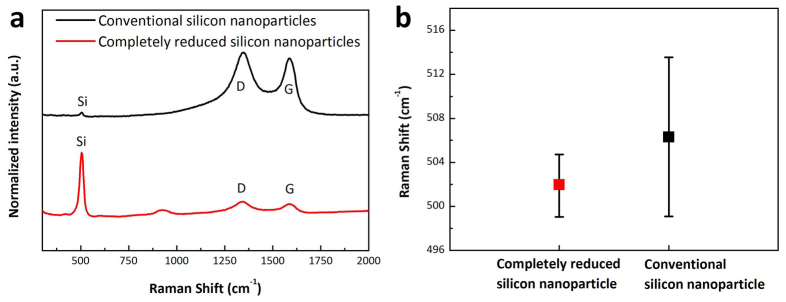
Raman analysis of completely reduced silicon nanoparticles (a) Raman spectra and (b) standard deviation of the silicon Raman peak positions of completely reduced silicon nanoparticles (red line) and conventional silicon nanoparticles (black line).

Raman spectroscopy was also employed to provide additional evidence that complete reduction takes place when mesoporous silica is employed in the reduction process. Two samples were analyzed following hydrofluoric acid treatment. The first contained silicon particles prepared by utilizing a conventional silica composite and the second used completely reduced silicon nanoparticle from mesoporous silica. The Raman spectra of the first material (Figure 5a) contains a very small peak corresponding to silicon at 504 cm^−1^ and two prominent peaks for graphene oxide at 1355 cm^−1^(D) and 1579 cm^−1^(G). However, the Raman spectrum of completely reduced silicon nanoparticles after HF treatment (Figure 5a) contains a very strong peak for silicon along with rGO peaks. The results clearly show that silicon particles produced by using the conventional method detach from rGO sheets because of the existence of silica residue between silicon and rGO sheets. Indeed, the graphene layer has strong van der Waals forces and, thus, it is easily re-stacked if no interlayer materials such as silicon nanoparticles exist^12^. However, when the mesoporous silica based method is employed, the absence of residual silica enables silicon to remain on the rGO sheets after HF treatment. This finding is in good agreement with the results of analysis of SEM images (see Supplementary Fig. S5 online), which show that the conventional reduction method generates aggregated rGO sheets after HF treatment. In contrast, completely reduced silicon nanoparticles on rGO sheets have two dimensional sheet-like morphology after HF treatment because silicon nanoparticles prevent re-stacking of rGO sheets (see Supplementary Fig. S5b online).

In addition, the standard deviation of Raman peak positions of silicon in the two samples were inspected (Figure 5b) to evaluate the uniformity of resulting silicon nanoparticles. Generally, the Raman spectrum of crystalline silicon contains one sharp peak at 520 cm^−1^ while spectrum of nano-crystalline silicon has this peak shifted to below 520 cm^−1^ 27,28 in a particle size dependent manner^27^. The narrower standard deviation of the silicon peak in the Raman spectrum of completely reduced silicon nanoparticles indicates that they have a relatively small and more uniform particle size (Figure 5b), a finding that agrees with estimate made from analysis of the TEM images (Figure 2c). The results demonstrate that the open cylindrical vertical pore nature of mesoporous silica leads to complete reaction with magnesium to create uniform and very small silicon nanoparticles that do not contain impurities such as silica and magnesium derivatives.

**Figure 6:**
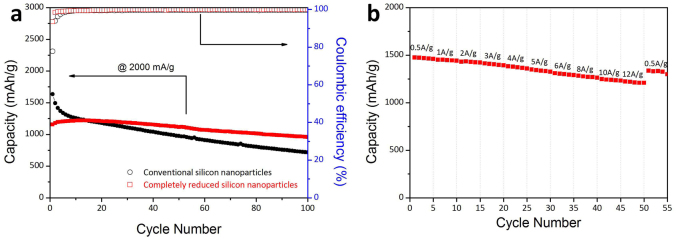
Electrochemical characteristics. (a) The cycling performances and coulombic efficiencies of unit cells fabricated using completely reduced silicon nanoparticles (red dot) and commercial silicon nanoparticles (black square) at 2 A/g, (b) rate capability of completely reduced silicon nanoparticle electrode measured at a series of current rates.

To demonstrate the applicability of the *ca.* 10 nm scale silicon nanoparticles on rGO sheets produced by using the new approach, we explored their use as an anode for Li ion batteries in the coin-type half-cells. We anticipated that the silicon nanoparticles on the rGO sheets would enable Li^+^ ion access and electron transfer, and would accommodate the severe volume changes taking place during battery operation^29^,^30^. For comparison purposes, two silicon anodes were generated, one containing commercially available silicon particles (Alfar Aesar, average particle size ≤50 nm) and the other completely reduced silicon nanoparticles. The cycling performances and coulombic efficiencies of half-cells containing both anodes were evaluated (Figures 6a and see Supplementary Fig. S6 online). The results show that the capacity of commercial silicon nanoparticle anode drops rapidly over time and reaches a value of 715 mAh/g after 100 cycles, with an initial capacity retention of 43.7%. The relatively poor cycling performance of this anode is attributed to the dramatic changes in volume of silicon particles occurring during Li ion insertion and the extraction process, which leads pulverization of the electrode materials and breakdown of the electrically conductive network. In contrast, the capacity of the anode fabricated using completely reduced silicon nanoparticles increases in the initial 24 cycles and retains 100% capacity until 40 cycles. Moreover, this anode retains 82.8% (956.7 mAh/g) of its capacity following 100 cycles. It should be noted that this level of capacity retention is achieved without the need to employ an additional carbon coating process. The superior cycling performance of the anode comprised of completely reduced silicon nanoparticles can be attributed to the small particle size (*ca.* 10 nm), which enables the anode to endure volume changes during the operation.

In order to determine the integrity of the electrodes after battery cycling, the cells were disassembled and the anodes were examined (see Supplementary Fig. S7 online). Inspection of the SEM images of the commercial silicon nanoparticle anode shows that the overall morphological stability is poor, with some cracks appearing on the electrode surface. However, the integrity and morphology of the completely reduced silicon nanoparticle anode was preserved over 100 cycles. The high morphology stability indicates that the two dimensional shape of completely reduced and un-aggregated silicon nanoparticles enables excellent pathways for the electrolyte. Also, the retention of capacity is mainly a consequence of the sub 10 nm size of the silicon nanoparticles bound onto the rGO sheets. According to the results of previous studies, the fracture toughness of lithiated silicon nanoparticles is significantly improved when they are smaller than 20 nm^31^, and 10 nm sized silicon particles have a capacity retention of 81% after 40 cycles^32^.

The anode comprised of completely reduced silicon nanoparticles displays an excellent rate capability at different rates (Figure 6b). The cell was exposed to an increasing current rate from 0.5 A/g (1/3 C) to 12 A/g (8 C). Surprisingly, the completely reduced silicon nanoparticle anode exhibits very stable capacity even at a 8 C rate, which indicates that the discharging time is only 7.5 min with the capacity of up to 1231 mA/g (83.4% of initial capacity at 0.5 A/g(Figures 6b and see Supplementary Fig. S8 online). Furthermore, the electrochemical performance of the anode derived from completely reduced silicon nanoparticles is demonstrated by using the electrochemical impedance (EIS) measurements after cycling in (see Supplementary Fig. S9 online). The charge transfer resistance of completely reduced silicon nanoparticle anode, determined by analysis of a Nyquist plot, is much smaller than that of the anode created by employing commercial silicon nanoparticles, indicating that faster charge transfer^18^,^33^,^34^ takes place as a consequence of the existence of excellent electrolyte pathways associated with the two dimensional shape of completely reduced silicon nanoparticles and their direct contact with the rGO sheets.

## Discussion

**Figure 7:**
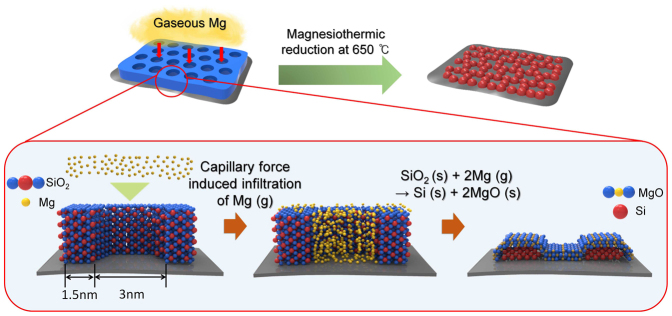
Proposed mechanism for complete reduction process. Illustration of the silicon nanoparticle formation using vertically aligned mesoporous silica channels.

As described above, a new method for carrying out complete magnesiothermic reduction of silica to produce silicon not containing undesired side products was developed. The process utilizes highly ordered vertically aligned mesoporous silica channels. The high efficiency of this reduction reaction can be attributed to interaction occurring between gaseous magnesium and silica that has a high surface area when arranged in this manner. As a result, efficient infiltration of gaseous magnesium takes place and gaseous magnesium diffuses into the vertically aligned, thin cylindrical mesopores. The mechanism of the complete reduction process is shown in Figure 7. The results of N_2_-sorption analysis show that the vertically aligned mesoporous silica channels have a high surface area of *ca.* 1030 m^2^/g, which provides a large number of reaction sites between silica and magnesium. Gaseous magnesium easily infiltrates into the nanoscale mesoporous silica channels of silica by capillary force. When heated to 650°C under a mixture of argon and hydrogen gas, gaseous magnesium driven by capillary forces diffuses into silica pore spaces. Moreover, the vertically aligned mesoporous cylinder channels provide optimum geometric confinement of gaseous magnesium^35^,^36^. These features enable complete reduction reaction. In other words, the mesoporous silica structure not only increases the number of reaction sites, it also causes geometric constraints^37^. Last but not least, the thin silica mesopore walls (*ca.* 1.5 nm) might also contribute to the complete reduction of silica.

In summary, the study has resulted in the development of a new, magnesiothermic reduction based method for fabrication of high quality of ca. 10 nm sized silicon nanoparticles on rGO sheets. The vertically aligned mesoporous silica channels effectively converted into silicon nanoparticles without any residues during the magnesiothermic reduction due to high surface area, confinement of gaseous magnesium, and thin mesopore walls. In addition, we have shown that the silicon nanoparticles (*ca.* 10 nm) on rGO sheets provide very stable electrochemical performance for Li-ion battery anode without further steps (carbon coating, HF etching) to handle its volume expansion and charge transfer problem. The simplicity and cost-effectiveness of the new method, which produces very small-sized nanostructures with the help of mesoporous templates, is advantageous for its potential use in a variety of promising applications.

## Methods

### Preparation of GO

Graphene oxide was synthesized using a modification of Hummer's method. Graphite powder (1 g, Aldrich) was added to sulfuric acid (98%, 30 mL). Then, potassium permanganate (3.5 g) was gradually added to the solution with vigorous stirring for 10 min. After stirring at 35°C for 2 h, the solution was cooled in an ice bath and diluted with deionized water (200 mL). Following 1 h of stirring, hydrogen peroxide (100 mL) was added to the resolution, which was then filtered and the filtrate was washed several times with hydrochloric acid (10%), and the solid GO dried under a vacuum at room temperature for 12 h.

### Synthesis of completely reduced silicon nanoparticles

Mesoporous silica on GO sheets (mSiO_2_-sheets) was synthesized by using a modification of the method described by Ozin's group^13^. A GO (0.1 g) dispersion in distilled water (200 mL) was added to cetyltrimethylammonium chloride (CTACl, 14.5 g) followed by stirring and sonication. Then 1 M NaOH (3 mL) was added to adjust the pH to 11.7. The mixture was stirred at 80°C for 90 min, cooled to room temperature (RT), and TEOS (0.2 g) was added dropwise to the solution. The mixture was stirred at 80°C for 24 h. The as-synthesized sample was collected by vacuum filtration and washed by diluted hydrochloric acid solution and then calcinated at 400°C in argon at a rate of 1.7°C/min and then held at 400°C for 4 h. The mSiO_2_-sheets (0.6 g) were positioned on the crucible (15 ml) and covered evenly with magnesium flake. The Mg:mesoporous silica weight ratio was 0.8:1. The crucible was placed in a tube furnace at 680°C for 3 h under an Ar (500 sccm) and H_2_ (50 sccm) atmosphere. The resulting sample was washed with 1 M hydrochloric acid and then vacuum filtered. The control sample of silica not containing pores was synthesized by using the method described by Liu's group^12^. Accordingly, 5 mL of TEOS was added to 85 mL of ethanol with stirring. Then, 7.4 g of GO solution (1 wt% in water), 100 mL of ethanol, and 5 mL of ammonium hydroxide are added and stirred for 6 h and aged for 8 h at room temperature.

### Material characterizations

The samples were characterized by using X-ray diffraction (XRD, Rigaku D/Max-2500(SWXD) and Rigaku D/Max-RB (12 KW)) using Cu Kα radiation (λ = 0.15406 nm), scanning electron microscopy (SEM, FEI Nova230), a transmission electron microscope (TEM, Philips Technai F20), Raman spectroscopy (Horiba Jobin Yvon ARAMIS) using Ar ion CW Laser (514.5 nm), X-Ray photoelectron spectroscopy (XPS, Thermo VG Scientific Sigma Probe), and Fourier-transform infrared spectroscopy (FT-IR, Bruker Alpha-P). N_2_-sorption was carried out at 77 K by a Tristar 3000, Micromeritics (U.S.A).

### Electrochemical characterization

Electrodes were fabricated using the completely reduced and commercial silicon nanoparticles (SiNPs, average particle size ≤50 nm, Alfar Aesar) as active materials, Super-P carbon (conductive agents) and polyacrylic acid (PAA, polymeric binder) in a weight ratio of 60:20:20. Coin-type electrochemical cells (2032) were assembled in an Ar-filled glove box and composed of the silicon based electrode, which were placed on copper foil, Li metal counter electrodes and a polyethylene separator (PE, Asahi Kasei), which was soaked with the electrolytes (1 M LiPF6 in ethylene carbonate/diethylene carbonate (EC/DEC, 50/50 by vol.%) containing 5 wt.% of fluoroethylene carbonate, PANAX ETEC). The assembled unit cells were cycled at current rates ranging from 100 mA g^−1^ to 12 A g^−1^ over potential ranges of 0.005–1.5 V (vs. Li/Li^+^) using a WBCS 3000 battery tester (Wonatech) at room temperature. Electrochemical impedance spectroscopy (EIS) measurements were conducted using Solartron 1400/1470E in the frequency range of 1 MHz–100 mHz.

## Author Contributions

K.H.K. and H.-T.J. proposed the mechanism and wrote the manuscript. K.H.K., K.M.C. and S.J.K. prepared the samples and performed characterization. D.J.L. and J.-K.P. involved the electrochemical analysis. H.-T.J. supervised the work.

## Supplementary Material

Supplementary Information Supplementary information

## References

[b1] Bao Z. *et al.* Chemical reduction of three-dimensional silica micro-assemblies into microporous silicon replicas. Nature 446, 172–175 (2007). 17344850 10.1038/nature05570

[b2] Myrhaug E. H. & Tveit H. Material balances of trace elements in the ferrosilicon and silicon processes. Electric Furnace Conference, Warrendale, PA, AIST (2000).

[b3] Lui N., Huo K., McDowell M. T., Zhao J. & Cui Y. Rice husks as a sustainable source of nanostructured silicon for high performance li-ion battery anodes. Sci. Rep. 3. 1919 (2013). 23715238 10.1038/srep01919 PMC3665957

[b4] Ibisate M., Golmayo D. & Lopez C. Silicon direct opals. Adv. Mater. 21, 2899–2902 (2009).

[b5] Wong D. P., Lien H.-T., Chen Y.-T., Chen K.-H. & Chen L.-C. Patterned growth of nanocrystalline silicon thin films through magnesiothermic reduction of soda lime glass. Green Chem. 14, 896 (2012).

[b6] Yoo J.-K., Kim J., Jung Y. S. & Kang K. Scalable fabrication of silicon nanotubes and their application to energy storage. Adv. Mater. 24, 5452–5456 (2012). 22865826 10.1002/adma.201201601

[b7] Yi R., Dai F., Gordin M. L., Chen S. & Wang D. Micro-sized Si-C composite with interconnected nanoscale building blocks as high-performance anodes for practical application in lithium-ion batteries. Adv. Energy Mater. 3, 295–300 (2013).

[b8] Magasinski A. *et al.* High-performance lithium-ion anodes using a hierarchical bottom-up approach. Nat. Mater. 9, 353–358 (2010). 20228818 10.1038/nmat2725

[b9] Wu H. *et al.* Stable cycling of double-walled silicon nanotube battery anodes through solid-electrolyte interphase control. Nat. nanotech. 7, 310–315 (2012). 10.1038/nnano.2012.35 22447161

[b10] Batchelor L., Loni A., Canham L. T., Hasan M. & Coffer J. L. Manufacture of mesoporous silicon from living plants and agricultural waste: an environmentally friendly and scalable process. Silicon 4, 259–266 (2012).

[b11] Zhu S. *et al.* Controlled fabrication of Si nanoparticles on grapheme sheets for Li-ion batteries. RSC Adv. 3, 6141–6146 (2013).

[b12] Xin X. *et al.* A 3D porous architecture of Si/graphene nanocomposite as high-performance anode materials for Li-ion batteries. J. Mater. Chem. 22, 7724–7730 (2012).

[b13] Wang Z.-M., Wang W., Coombs N., Soheilnia N. & Ozin G. A. Graphene Oxide-Periodic Mesoporous Silica Sandwich Nanocomposites with Vertically Oriented Channels. Acs Nano 4, 7437–7450 (2010). 21090789 10.1021/nn102618n

[b14] Barrett E. P., Joyner L. G. & Halenda P. P. The determination of pore volume and area distributions in porous substances. I. computations from nitrogen isotherms. J. Am. Chem. Soc. 73, 373–380 (1951).

[b15] Pierotti R. A. *et al.* Reporting physisorption data for gas/solid systems with special reference to the determinations of surface area and porosity. Pure Appl. Chem. 57, 603–619 (1985).

[b16] Brunauer S., Emmett P. H. & Teller E. Adsorption of gases in multimolecular layers. J. Am. Chem. Soc. 60, 309–319 (1938).

[b17] Yang S. *et al.* Graphene-based nanosheets with a sandwich structure. Angew. Chem. Int. Ed. 49, 4795–4799 (2010). 10.1002/anie.201001634 20512835

[b18] Kim K. H. *et al.* Sulfur infiltrated mesoporous graphene-silica composite as a polysulfide retaining cathode material for lithium-sulfur batteries. Carbon 69, 543–551 (2014).

[b19] Chen W., Fan Z., Dhanabalan A., Chen C. & Wang C. Mesoporous silicon anodes prepared by magnesiothermic reduction for lithium ion batteries. J. Electrochem. Soc. 158, A1055–A1059 (2011).

[b20] Chen D. *et al.* Reversible lithium-ion storage in silver-treated nanoscale hollow porous silicon particles. Angew. Chem. Int. Ed. 51, 2409–2413 (2012). 10.1002/anie.201107885 22287200

[b21] Jia H. *et al.* Novel three-dimensional mesoporous silicon for high power lithium-ion battery anode material. Adv. Energy. Mater. 1, 1036–1039 (2011).

[b22] Avila A., Montero I., Galan L., Ripalda J. M. & Levy R. Behavior of oxygen doped SiC thin films: An x-ray photoelectron spectroscopy study. J. Appl. Phys. 89, 212–216 (2001).

[b23] Kusunoki I. & Igari Y. XPS study of a SiC film produced on Si(110) by reaction with a C2H2 beam. Appl. Surf. Sci. 59, 95–104 (1992).

[b24] Račiukaitis G., Brikas M., Kazlauskienė V. & Miškinis J. Doping of silicon by carbon during laser ablation process. Appl. Phys. A. 85, 445–450 (2006).

[b25] Raider S. I., Flitsch R. & Palmer M. J. Oxide growth on etched silicon in air at room temperature. J. Electrochem. Soc. 122, 413–418 (1975).

[b26] Morita M., Ohmi T., Hasegawa E., Kawakami M. & Ohwada M. Growth of native oxide on a silicon surface. J. Appl. Phys. 68, 1272–1281 (1990).

[b27] Iqbal Z., Vepřek S., Webb A. P. & Capezzuto P. Raman scattering from small particle size polycrystalline silicon. Solid state Comm. 37, 993–996 (1981).

[b28] Hai N. H., Grigoriants I. & Gedanken A. Converting stöber silica and Mediterranean sand to high surface reaction under autogenic pressure at elevated temperature. J. Phys. Chem. C 113, 10521–10526 (2009).

[b29] Sun Y., Wu Q. & Shi G. Graphene based new energy materials. Energy Environ. Sci. 4, 1113–1132 (2011).

[b30] Xiang H. *et al.* Graphene/nanosized silicon composites for lithium battery anodes with improved cycling stability. Carbon 49, 1787–1796 (2011).

[b31] Park M. H., Kim K., Kim J. & Cho J. Flexible dimensional control of high-capacity Li-ion-battery anodes: from 0D hollow to 3D porous germanium nanoparticle assemblies. Adv. Mater. 22, 415–418 (2010). 20217731 10.1002/adma.200901846

[b32] Kim H., Seo M., Park M.-H. & Cho J. A critical size of silicon nano-anodes for lithium rechargeable batteries. Angew. Chem. Int. Ed. 49, 2146–2149 (2010). 10.1002/anie.200906287 20175170

[b33] Zhang H. & Braun P. V. Three-dimensional metal scaffold supported bicontinuous silicon battery anodes. Nano Lett. 12, 2778–2783 (2012). 22582709 10.1021/nl204551m

[b34] Kim K. H. *et al.* High quality reduced graphene oxide through repairing with multi-layered graphene ball nanostructures. Sci. Rep. 3 3251; 10.1038/srep03251 (2013). 24248235 PMC3832876

[b35] Davis M. E. Ordered porous materials for emerging applications., Nature 417, 813–821 (2002). 12075343 10.1038/nature00785

[b36] Na K. *et al.* Directing zeolite structures into hierarchically nanoporous architectures. Science 333, 328–332 (2011). 21764745 10.1126/science.1204452

[b37] Santiso E. E. *et al.* Adsorption and catalysis: The effect of confinement on chemical reactions., Appl. Surf. Sci. 252, 766–777 (2005).

