# Supplementary material for: Complete magnesiothermic reduction reaction of vertically aligned mesoporous silica channels to form pure silicon nanoparticles
Source: Sci Rep. 2015 Mar 11;5:9014. doi: 10.1038/srep09014 (PMC4355679; doi:10.1038/srep09014)
Supplement: Supplementary Information [file srep09014-s1.pdf]

Supplementary information

Complete magnesiothermic reduction reaction of vertically aligned mesoporous silica channels to form pure silicon nanoparticles

*Kyoung Hwan Kim, Dong Jin Lee, Kyeong Min Cho, Seon Joon Kim, Jung-Ki Park and Hee-Tae Jung**

Department of Chemical and Biomolecular Engineering (BK21+Program), Korea Advance Institute of Science and Technology (KAIST), Daejeon 305-701 (Korea)

*Corresponding author. E-mail: heetae@kaist.ac.kr

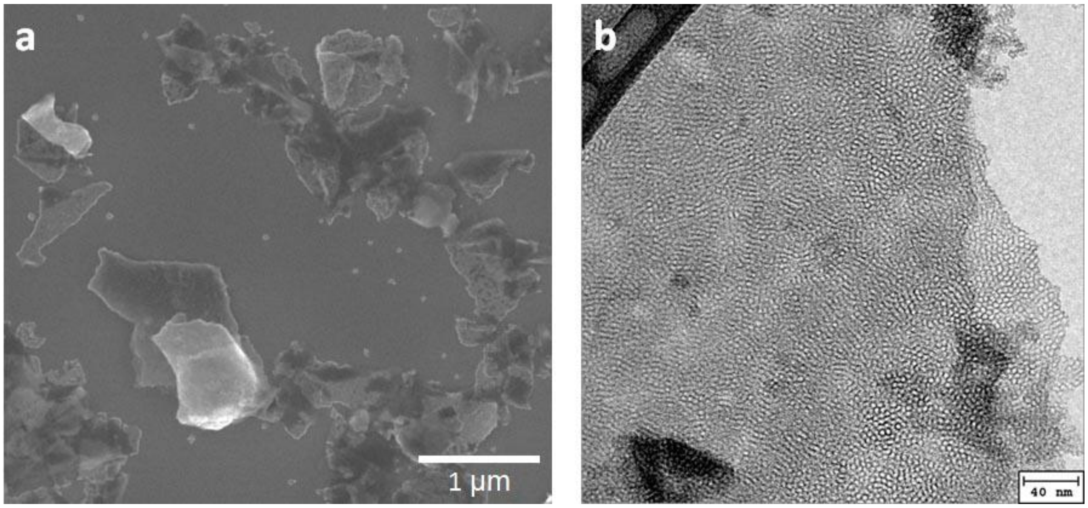

Figure S1. (a) SEM and (b) TEM image of the vertically oriented mesoporous silica channels on rGO sheets.

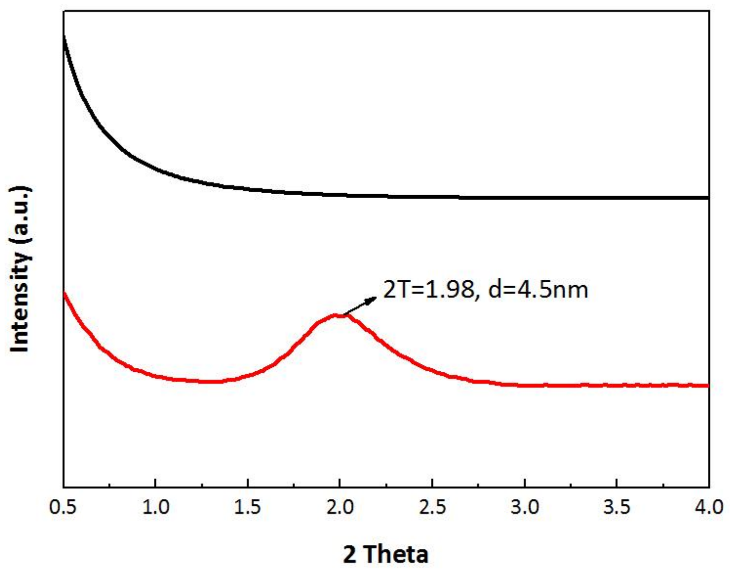

Figure S2. Small-angle X-ray scattering (SAXS) patterns of vertically aligned mesoporous silica channels (red line) and completely reduced silicon nanoparticle (black line)

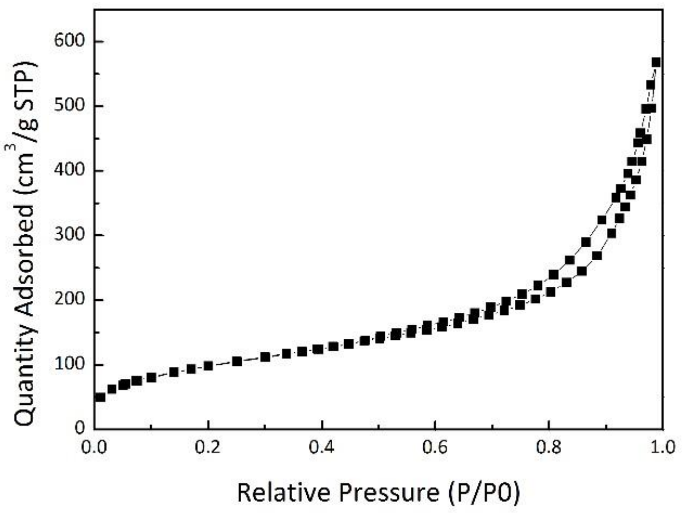

Figure S3. N_2-adsorption-desorption isotherms of **completely reduced silicon nanoparticle**.

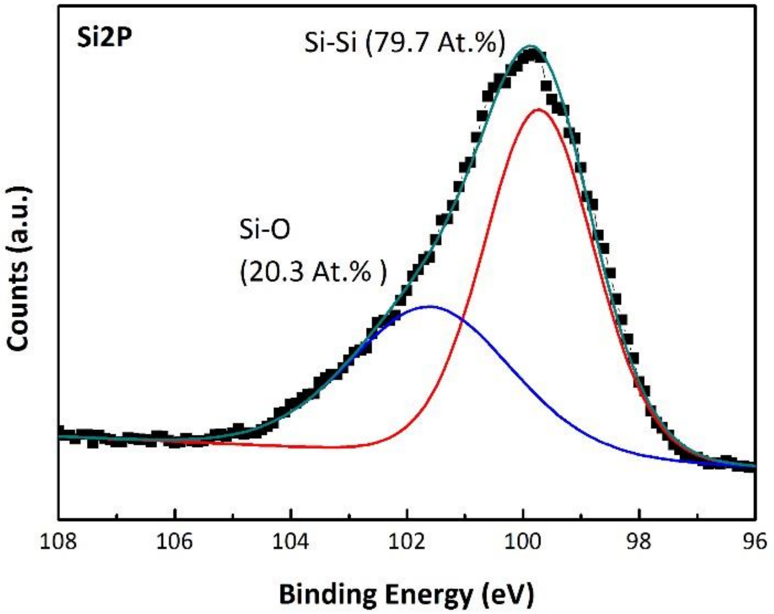

Figure S4. X-ray photoelectron spectroscopy (XPS) spectrum of Si2p of silicon nanoparticles prepared by using conventional silica composite after removing the residual silica by HF treatment of silicon particles.

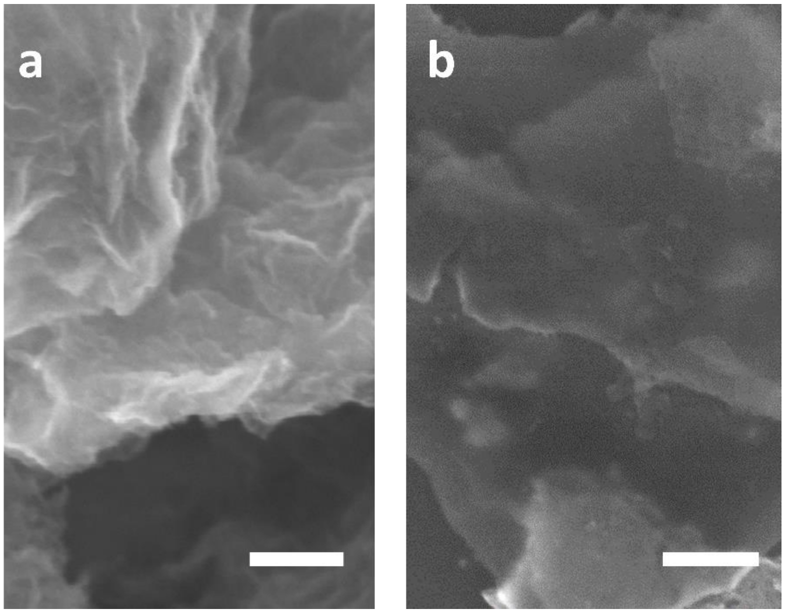

Figure S5. SEM image of (a) conventional silicon nanoparticles and (b) completely reduced silicon nanoparticles after HF treatment; scale bar, 400 nm.

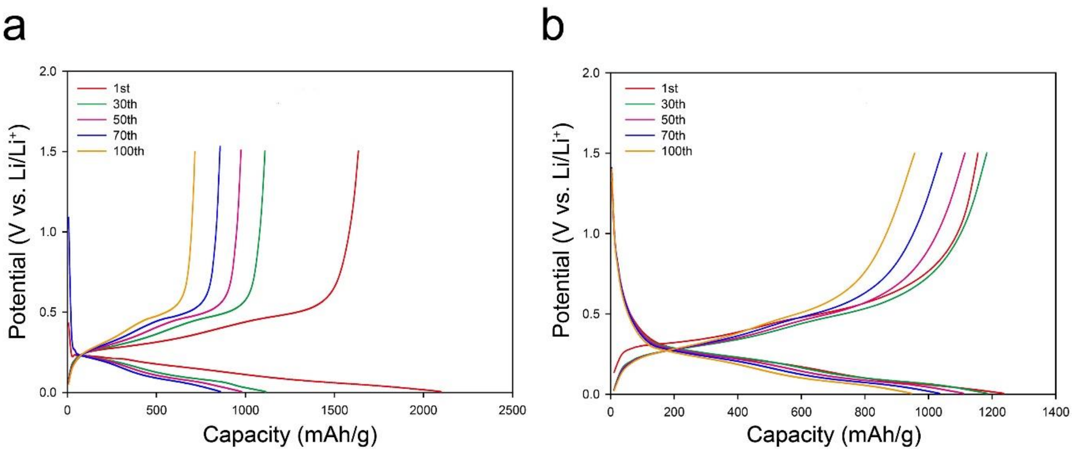

Figure S6. Charge-discharge voltage profiles of the (a) commercial silicon nanoparticle and (b) completely reduced silicon nanoparticle electrodes during cycles.

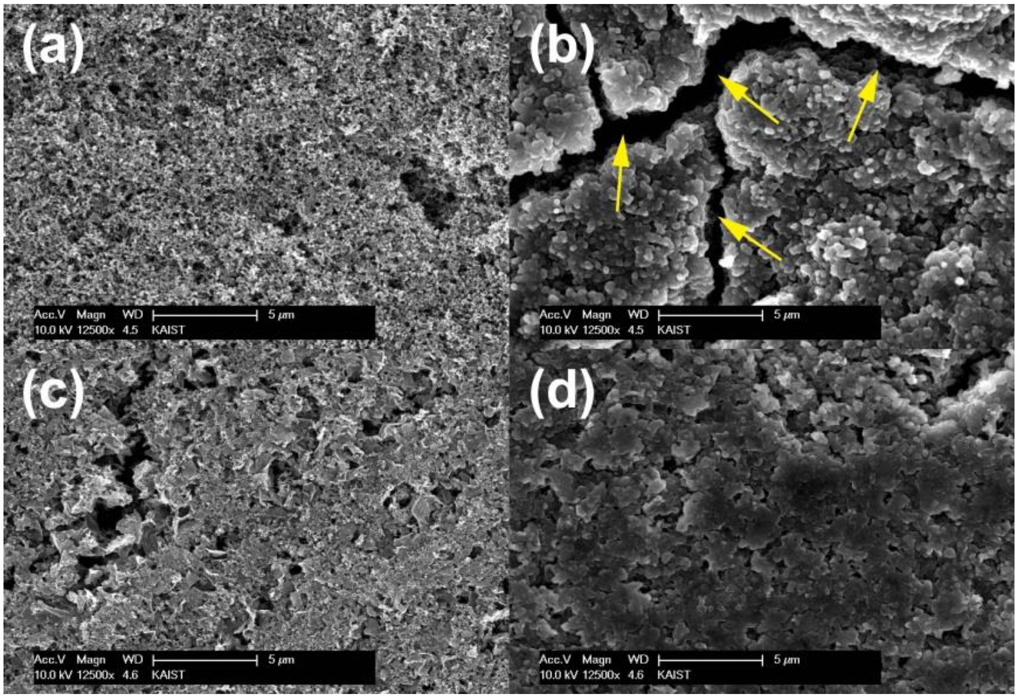

Figure S7. SEM images of the commercial silicon nanoparticle electrode (a) before cycling, (b) after 100 cycles (arrows indicate the crack formation of commercial silicon nanoparticle anode) and the completely reduced silicon nanoparticle electrode (c) before cycling, (d) after 100 cycles.

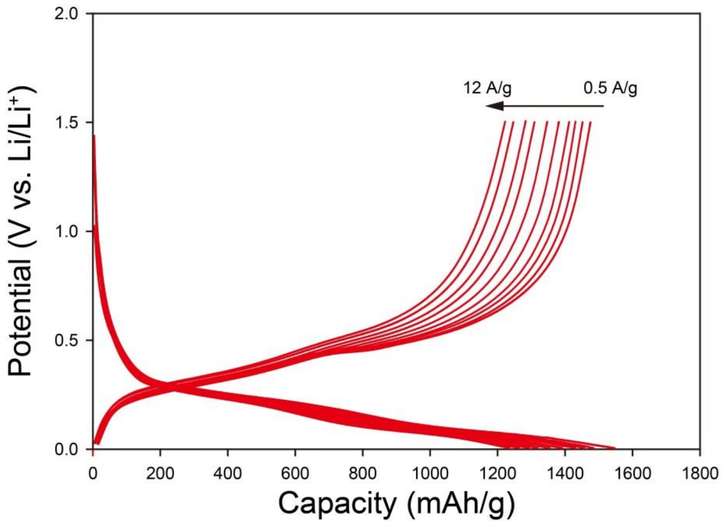

Figure S8. Charge-discharge voltage profiles of the completely reduced silicon nanoparticle cells at different rates

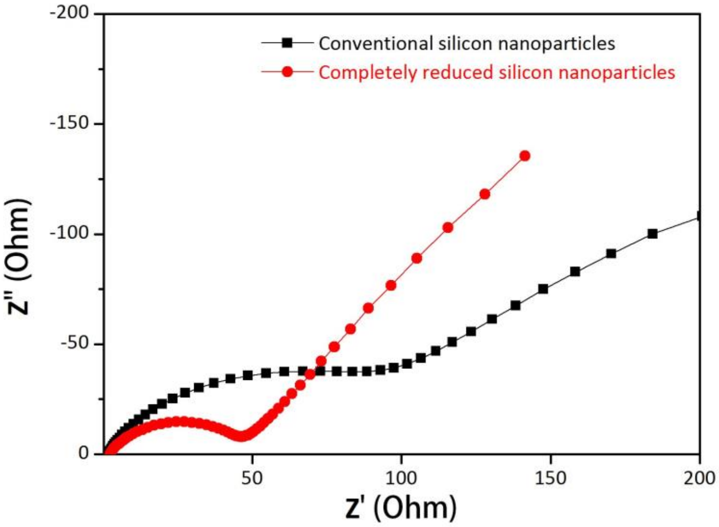

Figure S9. Impedance spectra of the unit cells after delithiation in the 100 cycles.
